# Freshwater Quality Criteria for Typical Quinolone Antibiotics: Norfloxacin, Enrofloxacin, and Ciprofloxacin

**DOI:** 10.3390/toxics14100864

**Published:** 2026-09-29

**Authors:** Qingyuan Pei, Bingjin He, Bin Zhu, Nannan Huang, Bin Shan, Weiqiang Tan

**Affiliations:** 1 School of Environmental & Municipal Engineering, Qingdao University of Technology, Qingdao 266520, China; yuanqingpei404@gmail.com (Q.P.); h15589643979@163.com (B.H.); zb19506132267@163.com (B.Z.); shanbin@qut.edu.cn (B.S.); 2 Chinese Research Academy of Environmental Sciences, Beijing 100012, China

**Keywords:** freshwater, water quality criteria, quinolone antibiotics, species sensitivity distribution, China

## Abstract

As a broad-spectrum antimicrobial agent, quinolones are widely used in animal husbandry. When applied to animals, 60–90% of antibiotics will be excreted in the form of the original drug or metabolites by animal excretion, contaminated soil, surface water, and ground water, affecting the normal life activities of plants, animals and microorganisms, and ultimately affecting human health through the food chain. Since some quinolone antibiotics are synthetic antibacterial agents and are used in large quantities, this paper discusses the technical routes, formulation processes, and results of three typical quinolone antibiotics. According to the existing technical guidelines in China, the optimal fitting model was used to derive the short-term water-quality criteria (SWQC) of norfloxacin as 0.019 mg/L and the long-term water-quality criteria (LWQC) as 0.0008 mg/L; the SWQC of enrofloxacin as 8.685 mg/L and the LWQC as 0.0625 mg/L; and the SWQC of ciprofloxacin as 0.5 mg/L and the LWQC as 0.106 mg/L.

## 1. Introduction

Antibiotics are extensively applied in human medicine, livestock production, and aquaculture, playing a critical role in disease control and productivity enhancement [1]. However, their widespread and continuously increasing use has raised growing concerns regarding the ecological risks associated with their environmental residues [2]. Aquatic systems serve as the primary sinks and transmission pathways for environmental antibiotics [3]. In these systems, antibiotic residues can disrupt microbial community structure and function [4,5,6], promote resistance selection and propagation [7,8], and induce toxicity in non-target organisms [9,10], including algal growth inhibition, invertebrate developmental toxicity, and endocrine disruption in fish [11,12], ultimately impairing ecosystem stability [13,14]. Furthermore, the antibiotic-driven dissemination of antibiotic resistance genes (ARGs) further amplifies ecological and human health risks [15,16]. Collectively, these impacts have intensified concerns about the potential effects of antibiotics on marine biota and ecosystem integrity.

Quinolone antibiotics are a class of broad-spectrum, highly effective, and low-toxicity antimicrobial agents, primarily used for the prevention and treatment of various bacterial infections in humans and animals. They are widely applied in fields such as healthcare, animal husbandry, agricultural planting, and aquaculture [17,18]. A WHO survey (1998) reported that annual quinolone consumption in the United States, Japan, South Korea, and the European Union was approximately 50 t as specialty products and 70 t as generic products, compared with 1350 t and 470 t, respectively, in China [19]. In 2018, global veterinary antibiotic consumption was approximately 81,000 tons, with an estimated dosage of 75.16–82.56 mg per kilogram of animal weight [20,21]. Ardakani [22] reported that in 2017, antimicrobial usage (AMU) in chickens, cattle, and pigs—comprising 93.75% of all food animals—amounted to 93,309 tonnes of active ingredients [23]. This figure is projected to rise by 11.5% to 104,079 tonnes by 2030. Following administration to humans or animals, 40–90% of antibiotics are excreted as parent compounds or metabolites and enter the environment via livestock and poultry manure [24]. In 2010, livestock and poultry manure discharged into the environment in China reached 4.5 billion t. Environmental monitoring has detected quinolone antibiotics in various environmental compartments, including water, sediments, and soil [25,26]. Notably, antibiotic concentrations in the water and soil surrounding some farms can reach abnormally high levels [27,28]. Currently, China widely employs high-performance liquid chromatography–tandem mass spectrometry (HPLC-MS/MS) for the detection of quinolone antibiotics [29,30]. Enrofloxacin is a compound that originates from a group of fluoroquinolones that are widely used in veterinary medicine as antibacterial agents (this antibiotic is not approved for use as a drug in humans) [31]. Norfloxacin is a third-generation quinolone antibacterial agent with inhibitory effects on bacteria. It is commonly used to treat enteritis, dysentery, respiratory tract infections, urinary tract infections, gonorrhea, and prostatitis caused by susceptible bacteria. However, this drug can delay bone formation in minors, thereby affecting their growth and development. Ciprofloxacin is also a third-generation quinolone antibacterial agent with broad-spectrum antibacterial activity and potent bactericidal effects. Its antibacterial activity against almost all bacteria is two to four times stronger than that of norfloxacin and enoxacin. However, it may cause side effects such as gastrointestinal reactions, central nervous system symptoms, and allergic reactions [32]. Basic information about the three quinolone antibiotics is shown in Table 1. This article demonstrates the technical route and the derived results for three representative quinolone antibiotics, which significantly support the future revision of water-quality criteria (WQC) for quinolone antibiotics in China.

In terms of derivation methods, the species sensitivity distribution method (SSD method) and the assessment factor method (AF method) are the most frequently used approaches for deriving antibiotic WQC. Since the antibiotic WQC values obtained by the SSD method and the toxicity percentile rank method are relatively close, the SSD method was selected. The SSD model is widely used; by integrating acute or chronic toxicity data from multiple species, it estimates the predicted no-effect concentration (PNEC) that protects a specified proportion of species (usually 95%) [33,34,35,36]. However, there are currently relatively few domestic studies on fluoroquinolone antibiotics. In this study, the commonly used norfloxacin (NOR), enrofloxacin (ENR), and ciprofloxacin (CIP) were selected as the research objects. The SSD method was used to calculate the WQC values for the three fluoroquinolone antibiotics, aiming to provide a reference for deriving WQC for fluoroquinolone antibiotics. On this basis, recommended reference values for SWQC and LWQC were derived.

## 2. Materials and Methods

### 2.1. Technical Approach to Criteria Development

#### 2.1.1. Technical Approach

**Figure 1:**
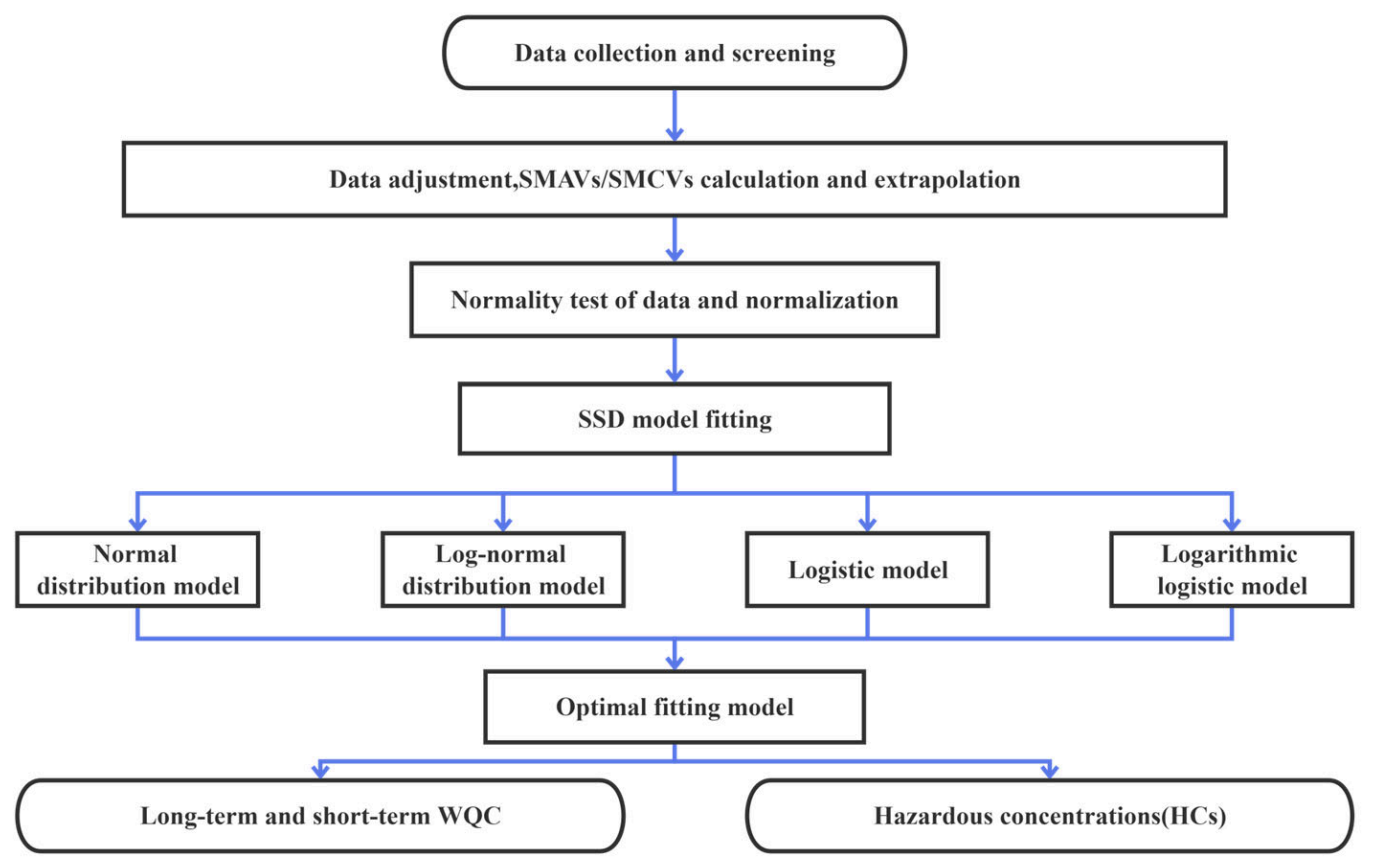
Technical pathway for developing national WQC for three typical quinolone antibiotics.

The technical route adopted in this study was based on the “Technical Guideline for Deriving Water Quality Criteria for Freshwater Organisms” issued by the Ministry of Ecology and Environment of China (MEE) [37], and is illustrated in Figure 1.

#### 2.1.2. WQCs Derivation Based on the SSD Model

The SSD approach was employed to derive WQCs, ensuring that the tested organisms collectively represented at least three trophic levels. Acute toxicity values (ATVs) were used to calculate acute response values (AVEs) at equivalent effect levels, while chronic toxicity values (CTVs) were used to compute chronic effect values (CVEs). All toxicity data were log-transformed prior to generating cumulative frequency distributions.

Four distribution models, the normal, log-normal, logistic and log-logistic distributions, were fitted to the SSD to estimate the hazardous concentration affecting 5% of species (HC_5_) [38]. A satisfactory model fit was indicated by a *p*-value greater than 0.05, and the model with the lowest root mean square error (RMSE) was selected for subsequent WQC derivation. The WQCs derived from HC_5_ (SHC_5_) based on acute toxicity data are called short-term WQCs (SWQC), while those derived from HC_5_ (LHC_5_) based on chronic toxicity data are called long-term WQCs (LWQC).

### 2.2. Collection and Screening of Toxicity Data

Toxicity data were collected from the published literature and publicly available toxicity databases. The English-language literature was retrieved from the Web of Science (https://www.webofscience.com), the Chinese-language literature from the CNKI database (https://www.cnki.net), and toxicity records from the ECOTOX database (https://cfpub.epa.gov/ecotox, accessed on 27 January 2026).

#### 2.2.1. Data Collection and Screening Rationales

Data collection and screening were performed in accordance with the guidelines. The exposure conditions adhered to the following principles:

(1) The experimental system (flow-through, semi-static, or static) was selected based on the physicochemical properties of the pollutant. Data from flow-through tests were preferred over semi-static tests, which in turn were preferred over static tests. For substances that are unstable in the test system, measured exposure concentrations were recorded. Only toxicity data for native Chinese freshwater organisms or for exotic species occurring in natural waters of China were collected.

(2) The test system satisfied the biological requirements of the test organisms. Water-quality parameters were maintained within stable ranges suitable for the test species. Dilution water was prepared according to standard test methods or from aerated tap water; distilled or deionized water was not used directly as dilution water.

(3) For acute toxicity data of aquatic animals (mainly LC_50_ and EC_50_), the exposure duration was approximately 24 h for rotifers, 48 h for daphnids and chironomids, and 96 h for other species. For chronic toxicity data of aquatic animals (mainly NOEC, LOEC, MATC, EC_10_, and EC_20_), the exposure duration was ≥48 h for rotifers and ≥21 days or covered a sensitive life stage (e.g., a fish early-life-stage test) for other species.

(4) For acute toxicity data of aquatic plants (mainly LC_50_ and EC_50_), the exposure duration was approximately 96 h. For chronic toxicity data of aquatic plants (mainly NOEC, LOEC, MATC, EC_10_, EC_20_, and EC_50_), the exposure duration was ≥21 days or spanned at least one generation.

(5) Toxicity data derived from measured concentrations were preferred over those based on nominal concentrations.

(6) For acute data of the same species, the priority order was LC_50_ > EC_50_. For chronic data of the same species, the priority order was EC_20_ > MATC > NOEC = EC_10_ > LOEC. Full life-cycle toxicity data were preferred over partial life-cycle data, and single-life-stage data were avoided whenever possible. For chronic plant data, LOEC was additionally prioritized over EC_50_.

(7) Life stages relatively sensitive to the target pollutant, such as larval and embryonic stages, were selected.

(8) Toxicity endpoint data for the same species and pollutant that differed by more than 10-fold were considered outliers; if the cause of such discrepancies could not be resolved, all data for that species were discarded [37].

#### 2.2.2. Data Quality Evaluation

Data quality was evaluated and classified in accordance with the guidelines [38]. Based on the evaluation results, data were categorized as follows: fully reliable data (obtained using standard toxicity test methods), data of limited reliability (from tests not conducted according to standard methods but judged reliable by experts), unreliable data (data generated through processes lacking credibility, together with non-preferred data produced by integrating information from different sources, e.g., without chemical monitoring), and uncertain data (lacking sufficient experimental details to permit a reliability judgment). Among these, fully reliable data and data of limited reliability were considered acceptable for criteria derivation.

### 2.3. Toxicity Data Preprocessing

A model was constructed based on the physicochemical properties of the pollutant and the results of toxicity studies. Water-quality parameters (e.g., temperature, hardness, pH, organic matter content, and suspended particulate matter content) or their transformed forms were used as the independent variable (x), and the corresponding toxicity values or their transformed forms were used as the dependent variable (y). A correlation regression analysis was then performed to determine the influence of water-quality parameters on pollutant toxicity. When this influence is significant and the underlying pattern is clearly established, an appropriate model must be established or applied to adjust the toxicity data.

#### 2.3.1. Calculation of the Acute Value for the Same Effect

Classify the species, and take the EC_50_ as the growth-type ATV and the LC_50_ as the survival-type ATV. Then, substitute them into Formula (1) to calculate the growth-type AVE and survival-type AVE for each species.

(1) $${AVE}_{i,k}=\sqrt[{m}]{{ATV}_{i,k,1}\times {ATV}_{i,k,2}\times \cdots \times {ATV}_{i,k,m}}$$

where AVE is the acute value for the same effect (μg/L or mg/L), i is the one species (dimensionless), k is the acute toxic endpoint (generally categorized into growth endpoints and survival endpoints, dimensionless), m is the number of ATVs, and ATV is the acute toxicity value (μg/L or mg/L).

The lower AVE between the growth-based AVE and the survival-based AVE is included in the subsequent calculation. If only one AVE is obtained, that value is directly used [37].

#### 2.3.2. Calculation of the Chronic Value for the Same Effect

For each species, chronic toxicity data (MATC, EC_10_, EC_20_, NOEC, LOEC, EC_50_, and LC_50_) were categorized by effect type (growth or reproduction) and assigned as growth-based or reproduction-based Chronic Toxicity Values (CTVs), while LC_50_ values were assigned as survival-based CTVs. These were then separately substituted into Formula (2) to calculate the species-specific growth-based CVE, reproduction-based CVE, and survival-based CVE.

(2) $${CVE}_{i,k}=\sqrt[{m}]{{CTV}_{i,k,1}\times {CTV}_{i,k,2}\times \cdots \times {CTV}_{i,k,m}}$$

where CVE is the chronic value for the same effect (μg/L or mg/L), i is the one species(dimensionless), k is the acute toxic endpoint (generally categorized into growth endpoints and survival endpoints, dimensionless), m is the number of CTVs, and CTV is the chronic toxicity value (μg/L or mg/L).

When multiple CVEs are available for a given species, the minimum value is retained for use in subsequent analyses; when only a single CVE value is available, it is used directly without further selection [37].

#### 2.3.3. Calculation of the Cumulative Frequency

The lgAVE and lgCVE values were sorted separately in ascending order, and R rank was assigned accordingly (the lowest toxicity value received rank 1, the second lowest rank 2, and so forth; in cases where two or more species exhibited identical toxicity values, they were arbitrarily assigned consecutive ranks). The acute and chronic cumulative frequencies (F_R_) were subsequently computed for each species following Formula (3) [39]:

(3) $$F_{R}=\frac{\sigma_{1}^{R}f}{N+1}\times 100\%$$

where F_R_ is the cumulative frequency, R is the rank of toxicity values (dimensionless), f is the frequency (number of species corresponding to toxicity rank R), and N is the total sum of all frequencies.

## 3. Results

### 3.1. Toxicity Data

A total of 94 acute data points were obtained in this study, covering 7 phyla, 16 families, and 21 species. A total of 79 chronic data points were obtained, covering 5 phyla, 13 families, and 18 species.

### 3.2. AVEs and CVEs

The three calculated typical quinolone antibiotics, AVEs and CVEs, for the baseline water-quality condition are listed in Table 2, Table 3 and Table 4.

A total of 69 groups of AVE and CVE data were obtained.

### 3.3. Normality and Normalization of the AVEs and CVEs

SSD model fitting was performed with the log-transformed acute toxicity values (lgAVE) and log-transformed chronic toxicity values (lgCVE) as the independent variable (x) and the corresponding cumulative frequency (F_R_) as the dependent variable (y). Four distribution models were applied: normal distribution, log-normal distribution, logistic, and log-logistic models. The fitting was carried out using the recommended software: “National Ecological Environment Criteria Calculation Software-Species Sensitivity Distribution Method, EEC-SSD, Version 1.0 [40]”.

Goodness-of-fit was evaluated based on the following parameters:

(a) Root mean square error (RMSE), with a value closer to 0 indicating higher precision of the model fit.

(b) The *p*-value from the Anderson-Darling (A-D) test, where *p* > 0.05 indicates that the model passes the test and conforms to the theoretical distribution.

Based on the goodness-of-fit results and professional judgment, among the models with *p* > 0.05, the one with the smallest RMSE was selected as the best-fit model. The curve obtained from the best-fit model showed good agreement with the data points used in the fitting, thereby ensuring that the WQC extrapolated from the SSD curve are statistically reasonable and reliable.

### 3.4. Model Results

**Figure 2:**
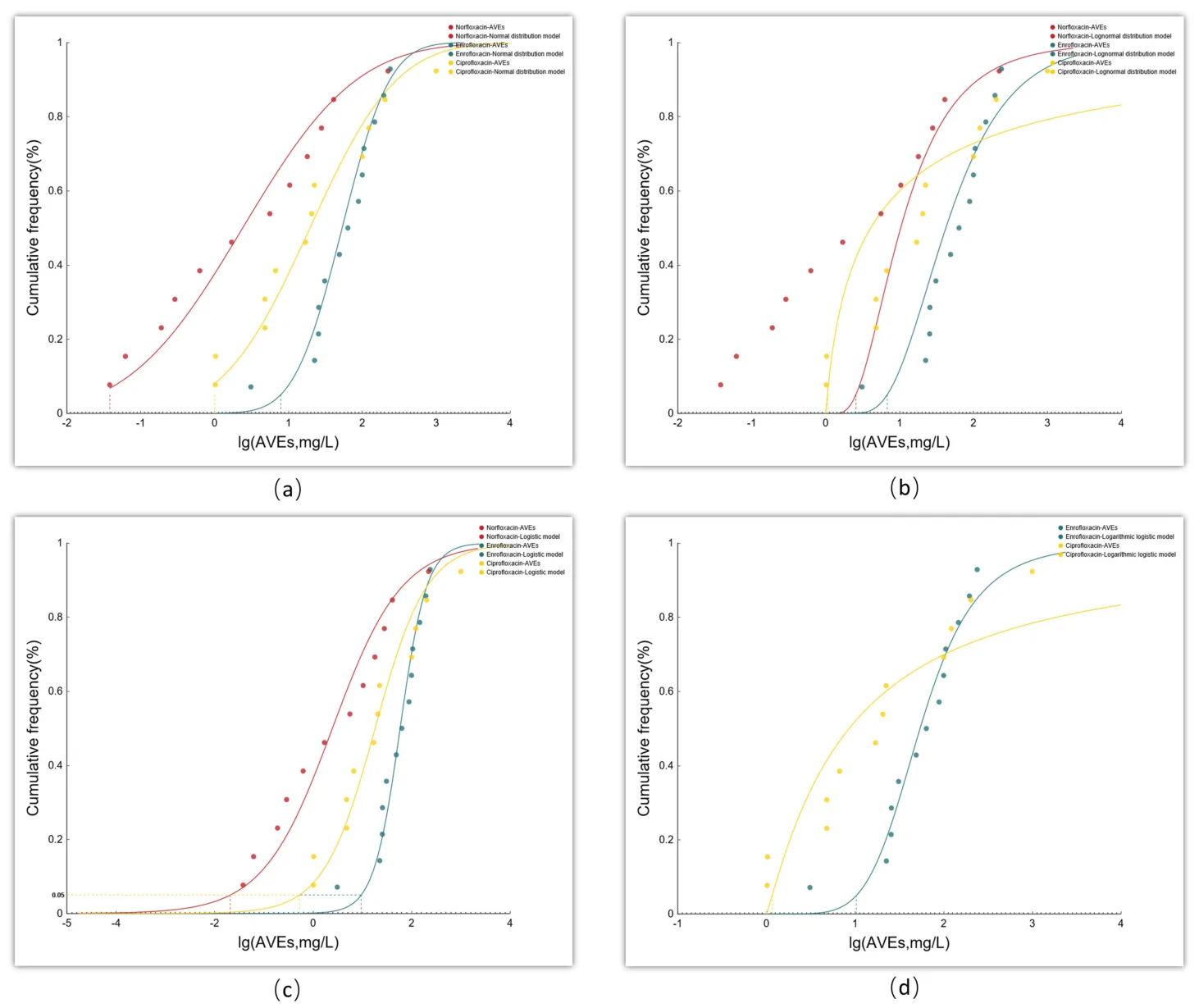
The fitting results of the four models for AVEs ((**a**): normal distribution model; (**b**): log-normal distribution model; (**c**): logistic model; (**d**): log-logistic model; The dashed lines in the figure are auxiliary lines used to indicate the HC_5_ of each antibiotic).

**Figure 3:**
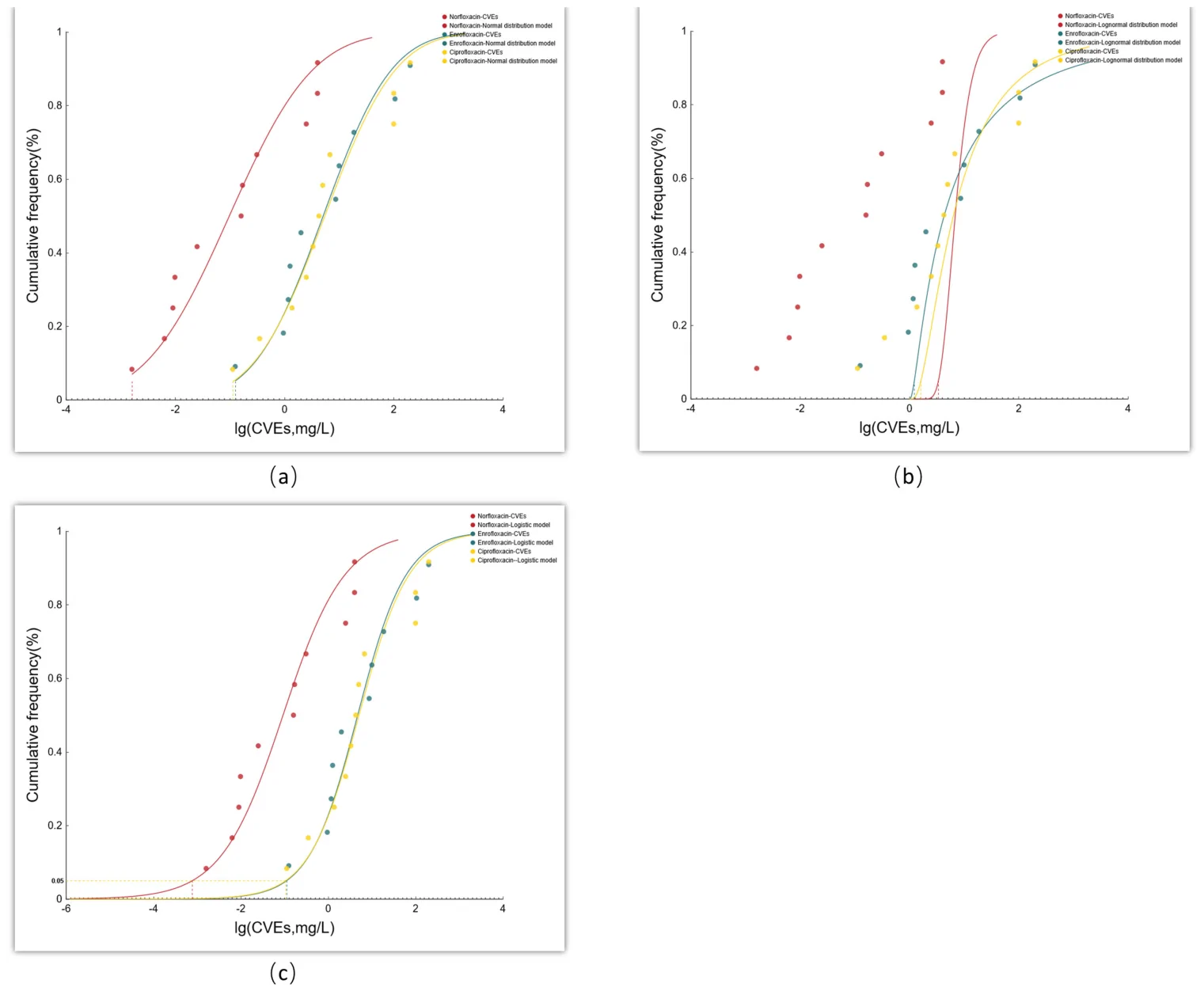
The fitting results of the four models for CVEs ((**a**): normal distribution model; (**b**): log-normal distribution model; (**c**): logistic model; The dashed lines in the figure are auxiliary lines used to indicate the HC_5_ of each antibiotic).

The 69 groups of AVEs and CVEs were fitted with four models (normal, log-normal, logistic and log-logistic). The fitting results for the acute toxicity values (AVEs) are presented in Figure 2, while those for the chronic toxicity values (CVEs) are presented in Figure 3.

### 3.5. Benchmark Setting

The benchmark extrapolation was conducted using Formulas (4) and (5) to obtain the short-term and long-term water-quality benchmarks, respectively.

(4) $$SWQC=\frac{{SHC}_{5}}{SAF}\;$$

where SWQC is the short-term water-quality criteria for aquatic organisms (μg/L or mg/L), SHC_5_ is the hazardous concentration for 5% of species, derived from the acute toxicity data using the fitted SSD curve (μg/L or mg/L), and SAF is the assessment factor applied to the short-term water-quality benchmarks for aquatic organisms (dimensionless).

(5) $$LWQC=\frac{{LHC}_{5}}{LAF}\;$$

where LWQC is the long-term water-quality criteria for aquatic organisms (μg/L or mg/L), LHC_5_ is the hazardous concentration for 5% of species, derived from the chronic toxicity data using the fitted SSD curve (μg/L or mg/L), and LAF is the assessment factor applied to the long-term water-quality benchmarks for aquatic organisms (dimensionless).

The assessment factor (AF) was determined based on factors such as the amount of data used for criteria derivation, the coverage of the tested species, and the distribution of the data-fitting, and it generally ranges from two to five. When the number of species represented by valid toxicity data exceeded 15, the AF was set to two; when the number of species was 15 or fewer, an AF of three was generally adopted [41,42]. In special circumstances (e.g., when algae accounted for more than 50% of the data or when the tail of the SSD curve showed poor fit), the AF was determined by expert judgment.

A self-audit of the criteria derivation process was conducted in accordance with the guidelines. The toxicity data used for criteria derivation met the following requirements simultaneously: (1) at least three trophic levels were covered, including producers, primary consumers, and secondary consumers; (2) at least three phyla and eight families of biological taxa were included [41]; (3) at least eight species were included, comprising a cyprinid fish (Osteichthyes), a non-cyprinid fish (Osteichthyes), zooplankton, and aquatic plants. Hence, the criteria derivation process did not deviate from the guidelines.

## 4. Discussion

The process data and the resultant water-quality benchmark values for the three typical fluoroquinolone antibiotics are summarized in Table 5, Table 6 and Table 7.

## 5. Conclusions

In this study, WQC were derived for norfloxacin, enrofloxacin, and ciprofloxacin—three quinolone antibiotics that are widely used and frequently detected in the environment. The derived SWQC were 0.019 mg/L for norfloxacin, 8.685 mg/L for enrofloxacin, and 0.5 mg/L for ciprofloxacin; the LWQC were 0.0008, 0.0625, and 0.106 mg/L, respectively. To date, no authoritative institution in China has issued freshwater environmental quality standards or WQC for norfloxacin, enrofloxacin, and ciprofloxacin. Moreover, research in China on the systematic derivation of freshwater aquatic-life WQC and human health benchmark values for these three typical quinolones remains relatively limited. Therefore, there is an urgent need to conduct research on WQC derivation, so as to provide theoretical support for the formulation of water environment standards for emerging pollutants in China and the implementation of relevant environmental governance measures.

## Figures and Tables

**Table 1:** Basic information about the three quinolone antibiotics.

Antibiotic	CAS Number	Molecular Formula	Molecular Weight	Chemical Structures
Norfloxacin	70458-96-7	C_16_H_18_FN_3_O_3_	319.33	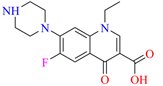
Enrofloxacin	93106-60-6	C_19_H_22_FN_3_O_3_	359.39	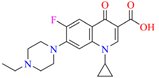
Ciprofloxacin	85721-33-1	C_17_H_18_FN_3_O_3_	331.34	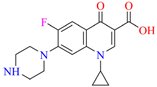

**Table 2:** Norfloxacin AVEs and CVEs of the baseline water-quality condition.

Species	AVEs (mg/L)	Species	CVEs (mg/L)
*Microcystis wesenbergii*	0.038	*Microcystis aeruginosa*	0.0016
*Microcystis aeruginosa*	0.062	*Microcystis wesenbergii*	0.0063
*Anabaena variabilis*	0.19	*Anabaena flosaguas*	0.009
*Anabaena flosaguas*	0.29	*Anabaena variabilis*	0.0098
*Synechococcus leopoliensis*	0.63	*Anabaena* sp.	0.025
*Nostoc commune* Vauch.	1.7	*Synechococcus leopoliensis*	0.16
*Anabaena* sp.	5.6	*Daphnia magna*	0.17
*Chlorella vulgaris*	10.4	*Nostoc commune* Vauch.	0.31
*Raphidocelis subcapitata*	18	*Ceriodaphnia dubia*	2.5
*Selenastrum capricornutum*	28.04	*Selenastrum capricornutum*	4.01
*Ceriodaphnia dubia*	40.98	*Chlorella vulgaris*	4.02
*Daphnia magna*	223.12		

**Table 3:** Enrofloxacin AVEs and CVEs of the baseline water-quality condition.

Species	AVEs (mg/L)	Species	CVEs (mg/L)
*Raphidocelis subcapitata*	3.1	*Chlorella pyrenoidosa*	0.125
*Chlorella vulgaris*	22.58	*Pseudokirchneriella subcapitata*	0.952
*Litopenaeus vannamei*	25.54	*Chrysosporum ovalisporum*	1.17
*Daphnia carinata*	25.74	*Chlorella vulgaris*	1.26
*Ceriodaphnia dubia*	30.98	*Ceriodaphnia dubia*	2
*Microcystis aeruginosa*	49	*Daphnia magna*	8.66
*Daphnia magna*	63.74	*Pimephales promelas*	10
*Scenedesmus quadricauda*	88.8	*Litopenaeus vannamei*	18.65
*Oryzias latipes*	100	*Barchydanio rerio var*	105.56
*Danio rerio*	105.56	*Moina macrocopa*	200
*Gobiocypris rarus*	146.99		
*Tetradesmus obliquus*	195.6		
*Moina macrocopa*	239.04		

**Table 4:** Ciprofloxacin AVEs and CVEs of the baseline water-quality condition.

Species	AVEs (mg/L)	Species	CVEs (mg/L)
*Lemna gibba*	1.02	*Eisenia fetida*	0.112
*Daphnia magna*	1.03	*Lemna gibba*	0.35
*Chironomus riparius*	4.8	*Selenastrum capricornutum*	1.37
*Lumbriculus variegatus*	4.8	*Ceriodaphnia dubia*	2.5
*Raphidocelis subcapitata*	6.7	*Oncorhynchus mykiss*	3.31
*Microcystis aeruginosa*	17	*Ceriodaphnia dubia*	4.27
*Chlorella vulgaris*	20.614	*Raphidocelis subcapitata*	5
*Ceriodaphnia dubia*	22.45	*Daphnia magna*	6.77
*Xenopus laevis*	100	*Xenopus laevis*	100
*Danio rerio*	122.47	*Brachydanio rerio*	100
*Lemna minor* L.	203	*Closterium ehrenbergii* Menegh	200
*Dugesia japonica*	1000		

**Table 5:** Model fitting results for acute WQC.

Acute Toxicity	Antibiotic	HC_5_	HC_10_	HC_25_	HC_50_	HC_75_	HC_90_	HC_95_	RMSE	*p* (A–D)
Normal distribution model	Norfloxacin	0.038	0.068	0.369	2.405	15.668	84.625	232.220	0.058	>0.05
	Enrofloxacin	14.246	19.200	31.608	54.992	95.697	157.543	212.276	0.075	>0.05
	Ciprofloxacin	1.000	1.488	5.762	25.936	116.735	452.064	1016.483	0.056	>0.05
Log-normal distribution model	Norfloxacin	2.560	3.156	5.000	10.371	29.950	116.708	337.443	\ *	>0.05
	Enrofloxacin	17.370	21.439	31.572	51.416	89.702	158.271	229.932	0.071	>0.05
	Ciprofloxacin	1.040	1.090	1.377	3.955	369.999	\	\	0.160	<0.05
Logistic model	Norfloxacin	0.021	0.070	0.417	2.475	14.700	87.315	293.341	0.064	>0.05
	Enrofloxacin	13.812	19.213	31.214	50.710	82.385	133.844	186.183	0.075	>0.05
	Ciprofloxacin	0.675	1.720	6.795	26.848	106.083	419.164	1067.212	0.061	>0.05
Logarithmic logistic model	Norfloxacin	\	\	\	\	\	\	\	\	\
	Enrofloxacin	16.873	21.405	31.510	48.705	79.537	138.182	209.258	0.076	>0.05
	Ciprofloxacin	1.110	1.254	2.030	9.162	1023.467	2.62 × 10^9^	2.89 × 10^20^	0.129	<0.05

* “\”indicates that no specific value was fitted.

**Table 6:** Model fitting results for chronic WQC.

Acute Toxicity	Antibiotic	HC_5_	HC_10_	HC_25_	HC_50_	HC_75_	HC_90_	HC_95_	RMSE	*p* (A–D)
Normal distribution model	Norfloxacin	0.002	0.003	0.015	0.097	0.636	3.436	9.432	0.073	>0.05
	Enrofloxacin	0.125	0.125	0.537	2.935	16.051	74.063	184.927	0.056	>0.05
	Ciprofloxacin	0.350	0.352	1.096	3.876	13.703	42.707	84.314	0.084	>0.05
Log-normal distribution model	Norfloxacin	3.397	3.869	4.961	6.904	10.283	15.783	21.164	\ *	>0.05
	Enrofloxacin	1.958	2.364	3.671	7.836	26.008	137.880	549.414	\	>0.05
	Ciprofloxacin	1.421	1.617	2.251	4.268	13.406	79.891	400.221	\	>0.05
Logistic model	Norfloxacin	0.001	0.003	0.015	0.092	0.551	3.289	11.088	0.076	>0.05
	Enrofloxacin	0.053	0.144	0.620	2.673	11.519	49.636	134.054	0.067	>0.05
	Ciprofloxacin	0.213	0.428	1.198	3.352	9.381	26.252	52.859	0.075	>0.05
Logarithmic logistic model	Norfloxacin	\	\	\	\	\	\	\	\	\
	Enrofloxacin	\	\	\	\	\	\	\	\	\
	Ciprofloxacin	\	\	\	\	\	\	\	\	\

* “\”indicates that no specific value was fitted.

**Table 7:** Summary of the results of WQC derivation.

Antibiotic	Criteria Category	Number of Species	WQC (mg/L)
Norfloxacin	SWQC	12	0.019
	LWQC	11	0.0008
Enrofloxacin	SWQC	7	8.685
	LWQC	5	0.0625
Ciprofloxacin	SWQC	10	0.5
	LWQC	6	0.106

## Data Availability

Data available in a publicly accessible repository.

## Abbreviations

The following abbreviations are used in this manuscript:

AF	Assessment factor
AMU	Antimicrobial usage
ARGs	Antibiotic resistance genes
ATV	Acute toxicity value
AVE	Acute value for the same effect
CIP	Ciprofloxacin
CTV	Chronic toxicity value
CVE	Chronic value for the same effect
EC_X_	x% effect concentration
ENR	Enrofloxacin
F_R_	Cumulative frequency
HC_X_	Hazardous concentration for x% of species
HPLC-MS/MS	High-performance liquid chromatography–tandem mass spectrometry
LAF	Assessment factor applied to the long-term water-quality benchmarks for aquatic organisms
LC_50_	Median lethal concentration
LHC_5_	Hazardous concentration for 5% of species, derived from the chronic toxicity data using the fitted SSD curve
LOEC	Lowest observed effect concentration
LWQC	Long-term water-quality criteria
MATC	Maximum acceptable toxicant concentration
MEE	Ministry of Ecology and Environment of China
NOEC	No observed effect concentration
NOR	Norfloxacin
RMSE	Root mean square error
SAF	Assessment factor applied to the short-term water-quality benchmarks for aquatic organisms
SHC_5_	Hazardous concentration for 5% of species, derived from the acute toxicity data using the fitted SSD curve
SSD	Species sensitivity distribution
SWQC	Short-term water-quality criteria for aquatic organisms
